# Membrane Charge and Packing Govern the Interfacial Insertion, Structure, and Dynamics of Ctx(Ile^21^)‑Ha and Its TOAC-Labeled Analogues in Biomimetic Liposomes

**DOI:** 10.1021/acsomega.6c07092

**Published:** 2026-10-17

**Authors:** Fadi S. S. Magalhães, Dayane S. Alvares, Rayssa P. R. Costa, Luis G. M. Basso, Eduardo F. Vicente

**Affiliations:** † Laboratory of Physical Sciences, Center of Sciences and Technology, State University of Northern Rio de Janeiro (UENF), Campos dos Goytacazes, Rio de Janeiro 28013-602, Brazil; ‡ Department of Physics, Institute of Biosciences, Letters, and Exact Sciences, São Paulo State University (UNESP), São José do Rio Preto, São Paulo 15054-000, Brazil; § Department of Animal Science, School of Agricultural and Veterinarian Sciences, São Paulo State University (UNESP), Jaboticabal, São Paulo 14884-900, Brazil; ∥ Department of Biosystems Engineering, School of Sciences and Engineering, São Paulo State University (UNESP), Tupã, São Paulo 17602-496, Brazil

## Abstract

Cationic antimicrobial peptides are promising candidates to combat antifungal resistance. However, a detailed understanding of their membranotropic activity is required for targeted applications and sequence engineering. Here, we investigated the native peptide Ctx­(Ile^21^)-Ha, derived from the Brazilian Cerrado tree frog *Boana albopunctata*, and its analogues site-specifically labeled with the paramagnetic amino acid TOAC (2,2,6,6-tetramethylpiperidine-*N*-oxyl-4-amino-4-carboxylic acid). Peptide interactions with biomimetic monolayers and liposomes mimicking human, fungal, and bacterial membranes were investigated using Langmuir monolayers, circular dichroism, and electron spin resonance (ESR). Anionic lipids, particularly palmitoyl-oleoyl-phosphatidylglycerol (POPG) and palmitoyl-oleoyl-phosphatidylserine (POPS), strongly enhanced peptide penetration into lipid monolayers and promoted substantial α-helical folding in large unilamellar vesicles (LUVs). The distinct monolayer responses of POPG- and POPS-containing membranes, despite their comparable α-helical contents, indicate that anionic headgroup chemistry modulates peptide–membrane interactions beyond electrostatic attraction. Conversely, cholesterol and ergosterol reduced peptide penetration and attenuated membrane-induced helix formation due to sterol-induced membrane packing. Site-directed ESR revealed lipid-dependent motional regimes for [TOAC^2^]­Ctx­(Ile^21^)-Ha and [TOAC^13^]­Ctx­(Ile^21^)-Ha, with the central region of the peptide becoming substantially more immobilized than the N-terminus upon membrane interaction. CD-derived membrane-association isotherms further showed that anionic membranes favor the coupled association/folding response, whereas comparison across the complementary techniques indicates that membrane association, peptide folding, and membrane penetration are mechanistically related but distinct steps in peptide–membrane interactions. Overall, the membrane activity of Ctx­(Ile^21^)-Ha is best described as a charge- and packing-modulated process, in which electrostatic interactions favor peptide binding and folding, whereas membrane order modulates membrane penetration and local peptide dynamics.

## Graphical abstract

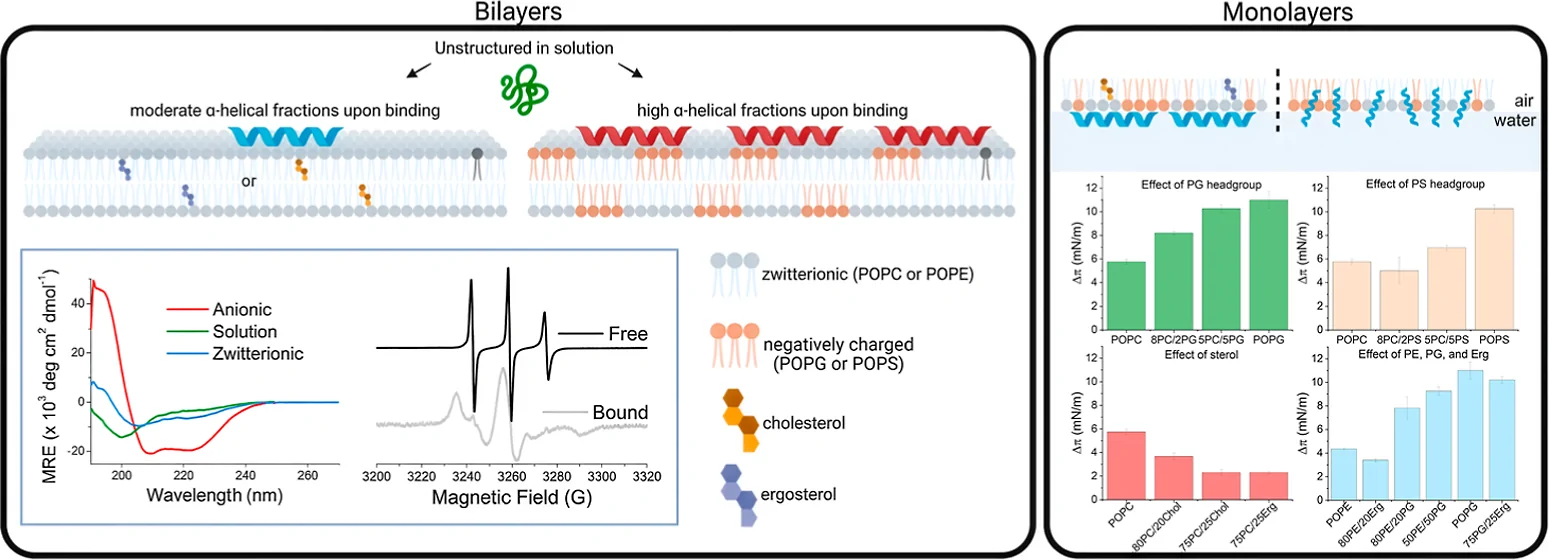

## Introduction

The global rise of infections caused by antimicrobial-resistant microorganisms has become a major challenge for clinical treatment, reinforcing the urgent need for novel therapeutic strategies. Consequently, cationic antimicrobial peptides (AMPs) have emerged as promising candidates due to their rapid and broad-spectrum activity, frequent membrane-directed mechanisms, evolutionary conservation as host-defense molecules, and potential to accelerate conventional antibiotic discovery pipelines.^1–6^

The biological activity of cationic AMPs is frequently associated with their ability to interact with and disrupt lipid membranes.^6–9^ However, intracellular targets and nonlytic mechanisms have also been reported for several families of cationic AMPs.^10–17^ These interactions often involve initial electrostatic attraction, followed by peptide adsorption, conformational rearrangement, and insertion into the lipid bilayer, ultimately leading to membrane destabilization, thinning, leakage, translocation, or pore formation.^18–27^ The antimicrobial activity for these molecules is highly modulated by membrane physicochemical properties, such as lipid composition, fluidity, surface charge, packing, intrinsic curvature, and headgroup chemistry, with microbial membranes typically enriched in anionic phospholipids.^28–33^

The incorporation of paramagnetic amino acids, such as TOAC has significantly advanced the study of peptide local dynamics.^34^ When strategically introduced into peptide sequences, TOAC acts as a conformationally constrained spin label, enabling a detailed investigation of peptide mobility, orientation, and depth of insertion in membrane environments through electron spin resonance (ESR) spectroscopy, providing site-specific structural information that is often inaccessible by conventional ensemble techniques.^35–37^ More recently, TOAC-labeled AMP analogues have also been explored as intramembrane radical sources for dynamic nuclear polarization nuclear magnetic resonance studies in lipid membrane environments, further expanding the applications of this paramagnetic amino acid in membrane biophysics.^38^ Combined with circular dichroism (CD) and Langmuir monolayer approaches, these techniques provide a powerful framework for membranotropic investigations, enabling mechanistic insights into how physicochemical differences in lipid envelopes modulate AMP activity across distinct organisms.

To better understand these mechanisms, biomimetic membrane systems, such as Langmuir monolayers and lipid vesicles, have been widely employed. These systems allow controlled investigation of peptide–membrane interactions under well-defined conditions, enabling the evaluation of peptide insertion, interface activity, membrane binding, lipid selectivity, and structural adaptation across distinct lipid environments.^39–42^ One cationic AMP of particular biotechnological interest is Ctx­(Ile^21^)-Ha, which is derived from the skin secretion of the tree frog *Boana albopunctata*. This linear peptide, with the primary sequence GWLDVAKKIGKAAFNVAKNFI-NH_2_, exhibits potent antimicrobial activity against both Gram-positive and Gram-negative bacteria, as well as fungi, while exhibiting a relatively low cytotoxicity toward mammalian cells.^43^ Consistent with this general selectivity profile, previous studies using TOAC-labeled Ctx­(Ile^21^)-Ha analogues, which were shown to retain antibacterial activity although their relative potencies depended on the labeling position, exhibited more favorable interactions with phosphatidylethanolamine-containing bacterial-like membrane models compared to sphingomyelin-containing mammalian-cell-like models. These studies also showed that the peptides adopt stable α-helical conformations in membrane-mimetic environments, including micelles and large unilamellar vesicles (LUVs).^44,45^

In this study, single-component, binary, and ternary lipid systems containing cholesterol (Chol), ergosterol (Erg), and phospholipids with distinct headgroups such as phosphatidylcholine (POPC), phosphatidylethanolamine (POPE), phosphatidylglycerol (POPG), and phosphatidylserine (POPS), were employed as simplified models representing selected physicochemical features of human, fungal, Gram-positive, and Gram-negative cell membranes, enabling a controlled investigation of the influence of membrane surface charge, phospholipid headgroup chemistry, and sterol content on peptide-membrane interactions. These biomimetic systems allow the evaluation of peptide insertion, orientation, interfacial activity, membrane binding, lipid selectivity, membrane perturbation, and structural adaptation across distinct lipid environments,^46–49^ thereby providing molecular-level insights into the lipid-dependent mechanisms underlying the membranotropic activity of the Ctx­(Ile^21^)-Ha scaffold, which remain poorly understood.

## Results and Discussion

### Interfacial Properties of Ctx­(Ile^21^)-Ha and Enhanced Perturbation of Anionic Lipid Monolayers

Surface activity experiments revealed the marked interfacial activity of Ctx­(Ile^21^)-Ha peptide, as evidenced by the concentration-dependent increase in surface pressure (π) observed upon peptide adsorption at the air–water interface (Figure 1A). The surface pressure increased progressively with peptide concentration until reaching a plateau at approximately 20 mN/m, consistent with values previously reported for highly surface-active cationic AMPs.^40,50^ Notably, this maximum was achieved at a low peptide concentration (∼0.7 μM), highlighting the peptide’s high propensity to adsorb at the air–water interface. The saturation observed near 20 mN/m suggests the formation of a densely packed interfacial layer, where further adsorption is limited.

**Figure 1:**
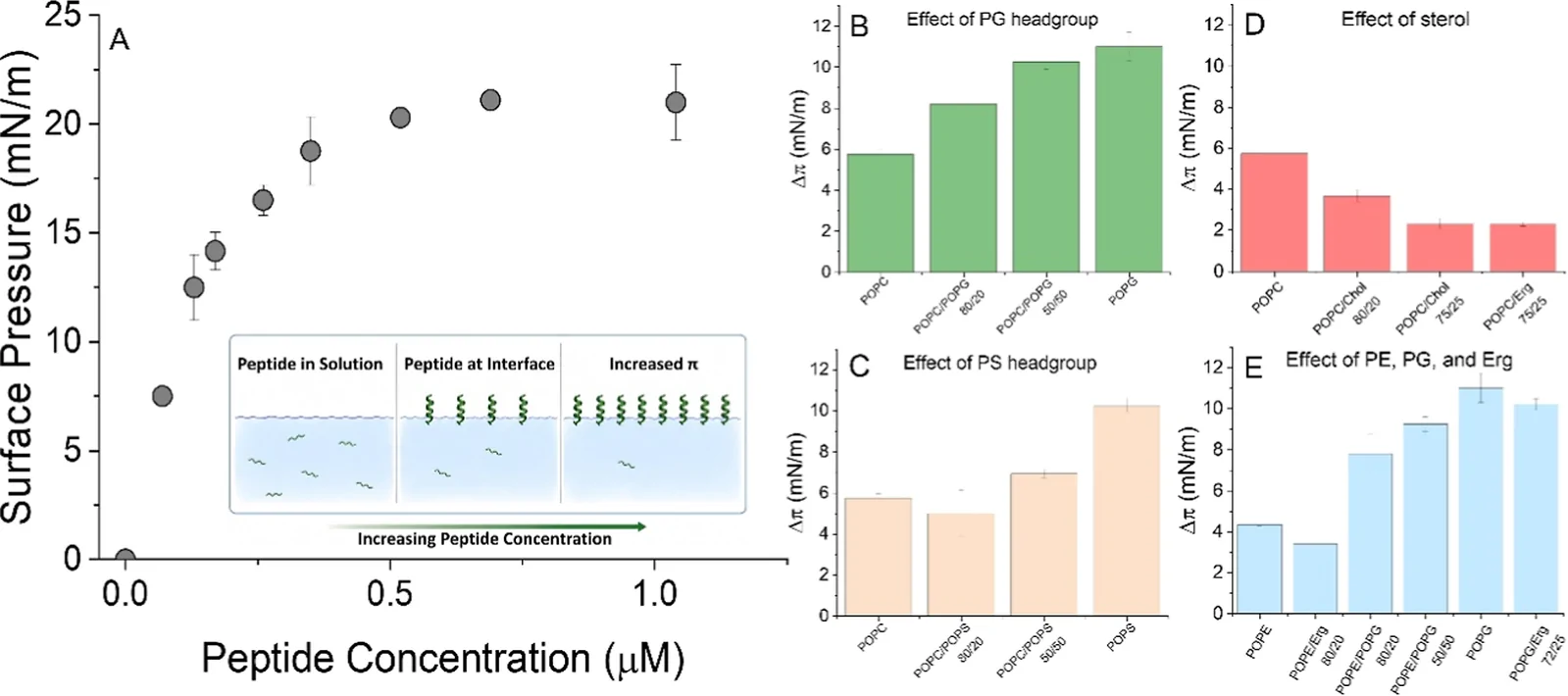
Surface activity and insertion of Ctx­(Ile^21^)-Ha into lipid monolayers. (A) Surface pressure (π) as a function of peptide concentration. The inset schematically illustrates the adsorption process: (1) peptide injection into the aqueous subphase, (2) peptide adsorption at the air–water interface, and (3) peptide accumulation at the interface leading to an increase in surface pressure. Panels (B–E) show peptide-induced changes in surface pressure upon insertion into lipid monolayers at an initial surface pressure of 30 mN/m, highlighting the effects of the PG headgroup (B), PS headgroup (C), sterol content (D), and PE, PG, and ergosterol-containing systems (E).

The ability of Ctx­(Ile^21^)-Ha to insert into lipid monolayers was further evaluated at an initial surface pressure of 30 mN/m, mimicking the lipid lateral packing density typically found in biological membranes. As shown in Figure 1B,C, the effect of negatively charged lipids on the peptide-induced increase in surface pressure depended on both the lipid headgroup and its mole fraction. Increasing POPG content produced a progressive increase in Δπ, whereas the effect of POPS was less pronounced at lower mole fractions and became more evident at higher POPS contents. For example, at 20 mol % anionic lipid, POPC/POPG monolayers exhibited a larger increase in surface pressure than POPC/POPS monolayers (∼8 mN/m versus ∼5 mN/m, respectively). These results indicate that net charge alone does not determine peptide-monolayer interactions and suggest an additional contribution from headgroup-specific physicochemical properties. PG and PS differ in headgroup size, hydration, hydrogen-bonding capacity, and interfacial organization, properties that have been shown to influence peptide–membrane interactions.^51,52^ At higher fractions of anionic lipids, larger peptide-induced increases in surface pressure were observed (∼10 mN/m), consistent with an increasing contribution of electrostatic interactions.

The presence of sterols modulated peptide penetration into lipid monolayers. Cholesterol strongly reduced peptide-induced increase in surface pressure (e.g., POPC/Chol 80:20 ∼3.7 mN/m; POPC/Chol 75:25 ∼2.4 mN/m) compared to pure POPC (∼5.8 mN/m) (Figure 1D). This behavior is consistent with its well-known condensing and acyl-chain-ordering effects, increasing lipid packing and mechanical resistance. Although cholesterol can also alter the organization and dynamics of the interfacial headgroup region, the present measurements do not provide direct information on peptide insertion depth; nevertheless, the smaller peptide-induced increase in Δπ indicates reduced perturbation of lipid packing under these conditions. A similar effect was observed in the presence of ergosterol (POPC/Erg 75:25 ∼2.3 mN/m) (Figure 1D). Interestingly, in PG-containing systems, ergosterol did not suppress insertion, showing values comparable to PG alone (Figure 1E). Similarly, systems such as POPE/POPG 80:20 and POPE/POPG 50:50 displayed responses comparable to the corresponding POPC/POPG mixtures (Figure 1E). These results indicate that the presence of POPG can partially counteract the reduction in peptide-induced surface-pressure changes associated with sterol incorporation and that, under these conditions, the effect of ergosterol on peptide insertion is less pronounced than that of cholesterol.

Taken together, these findings indicate that the interaction of Ctx­(Ile^21^)-Ha with lipid monolayers is governed by an interplay between electrostatic attraction and lipid packing properties. While anionic lipids generally promote peptide penetration into lipid monolayers, sterol-induced lipid condensation reduces this effect, particularly in zwitterionic POPC systems. The balance between these effects appears to contribute to the preferential interaction of the peptide with bacterial-like membrane models over mammalian membrane models, consistent with the established behavior of many cationic antimicrobial peptides.^53,54^

### Membrane Charge and Sterol Content Modulate Ctx­(Ile^21^)-Ha Folding and Membrane Association

Ctx­(Ile^21^)-Ha has been previously reported to undergo conformational changes from a predominantly disordered state in solution to more ordered structures upon interaction with different model membranes.^44,45^ However, those studies were performed either in micelles or in saturated phospholipid bilayers in the gel phase. Therefore, we revisited the membrane-induced conformational response of Ctx­(Ile^21^)-Ha using LUVs composed predominantly of unsaturated phospholipids in the fluid phase, aiming to better reproduce physiologically relevant membrane environments and to systematically investigate how membrane surface charge and lipid packing modulate peptide secondary structure and membrane association. Figure 2 illustrates the far-UV CD spectra of Ctx­(Ile^21^)-Ha in solution and in the presence of different membrane compositions.

**Figure 2:**
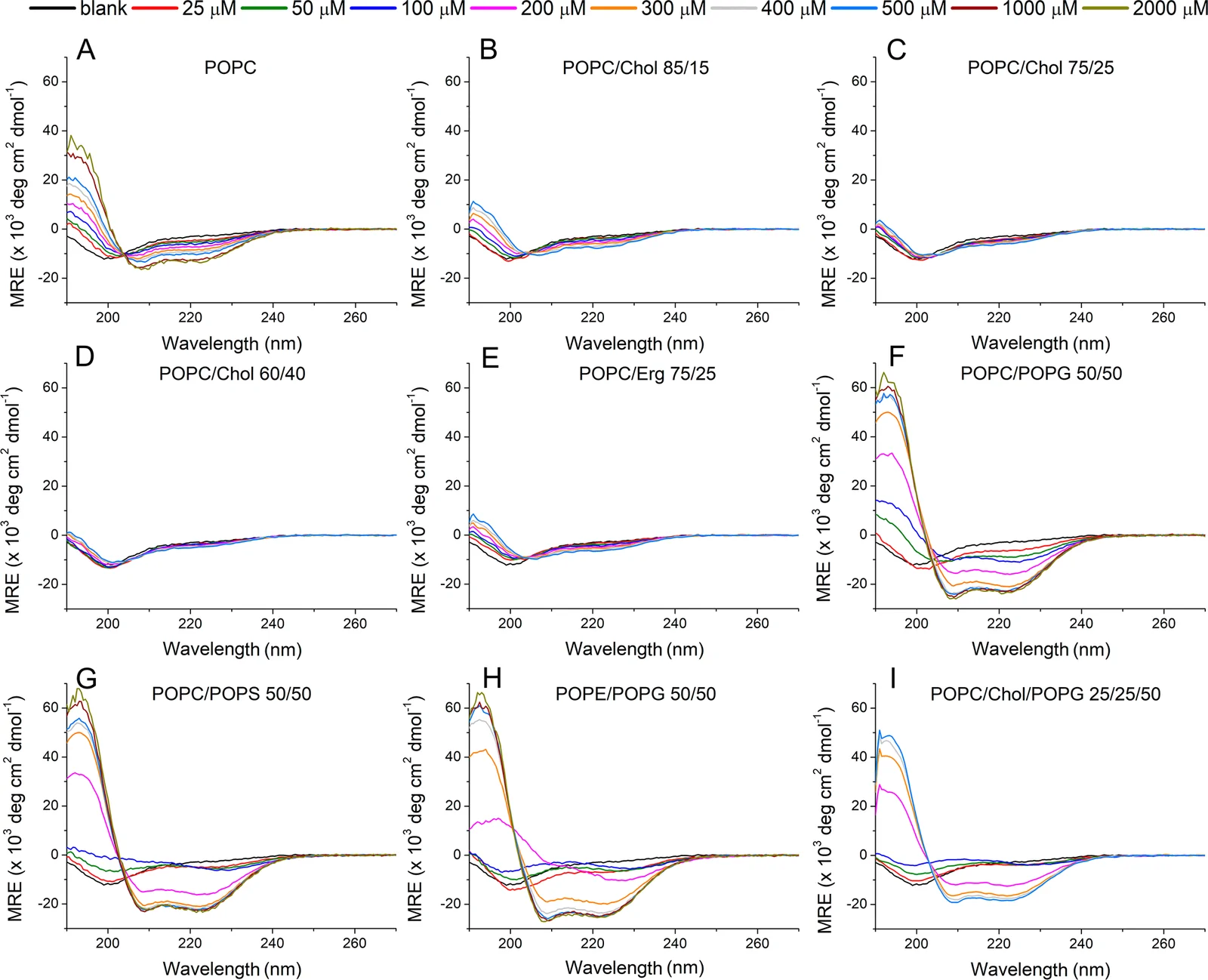
Far-UV CD spectra of 30 μM Ctx­(Ile^21^)-Ha in the absence (black line) and presence of increasing concentrations (25–2000 μM) of LUVs composed of (A) POPC, (B–D) POPC/Chol, (E) POPC/Erg, (F) POPC/POPG, (G) POPC/POPS, (H) POPE/POPG, and (I) POPC/Chol/POPG mixtures. All measurements were performed in 10 mM potassium phosphate (pH 7.0) at 25 °C.

In aqueous solution, Ctx­(Ile^21^)-Ha displays a predominantly disordered conformation, characterized by a strong minimum near 198–200 nm. Upon the addition of LUVs, several membrane systems progressively induced characteristic α-helical features, including negative bands near 208 and 222 nm and increased positive ellipticity below 200 nm, indicating membrane-induced peptide folding. Because the measured CD spectra may contain contributions from both free and membrane-associated peptide populations, the observed ellipticity represents a population-weighted conformational response. Consequently, the α-helical content of the membrane-associated state cannot be obtained directly from θ_222_ unless a limiting membrane-associated response is reached or its limiting ellipticity (θ_b_) can be reliably estimated. To resolve this contribution, the normalized ellipticity at 222 nm, θ_obs_/θ_0_, was plotted as a function of lipid concentration and fitted using the coupled membrane binding/folding model described in eq 2 (Figure 3). The model yields the limiting ellipticity of the membrane-associated state (θ_b_), the apparent membrane-association parameter (*K*_app_), and, when required, the Hill coefficient (*n*_H_). The α-helical content of the limiting membrane-associated state was then calculated from θ_b_ using the Luo–Baldwin formalism (eq 1).^55^ For systems in which a limiting response could not be reliably determined, the helicity calculated from θ_222_ at the highest lipid concentration tested represents an apparent ensemble-averaged value. The corresponding conformational and membrane-association parameters are summarized in Table 1. A spectrum recorded in 75% (v/v) trifluoroethanol (TFE), used as a helix-promoting reference, is shown in Figure S1 and corresponds to an estimated α-helical content of 92.4%.

**Figure 3:**
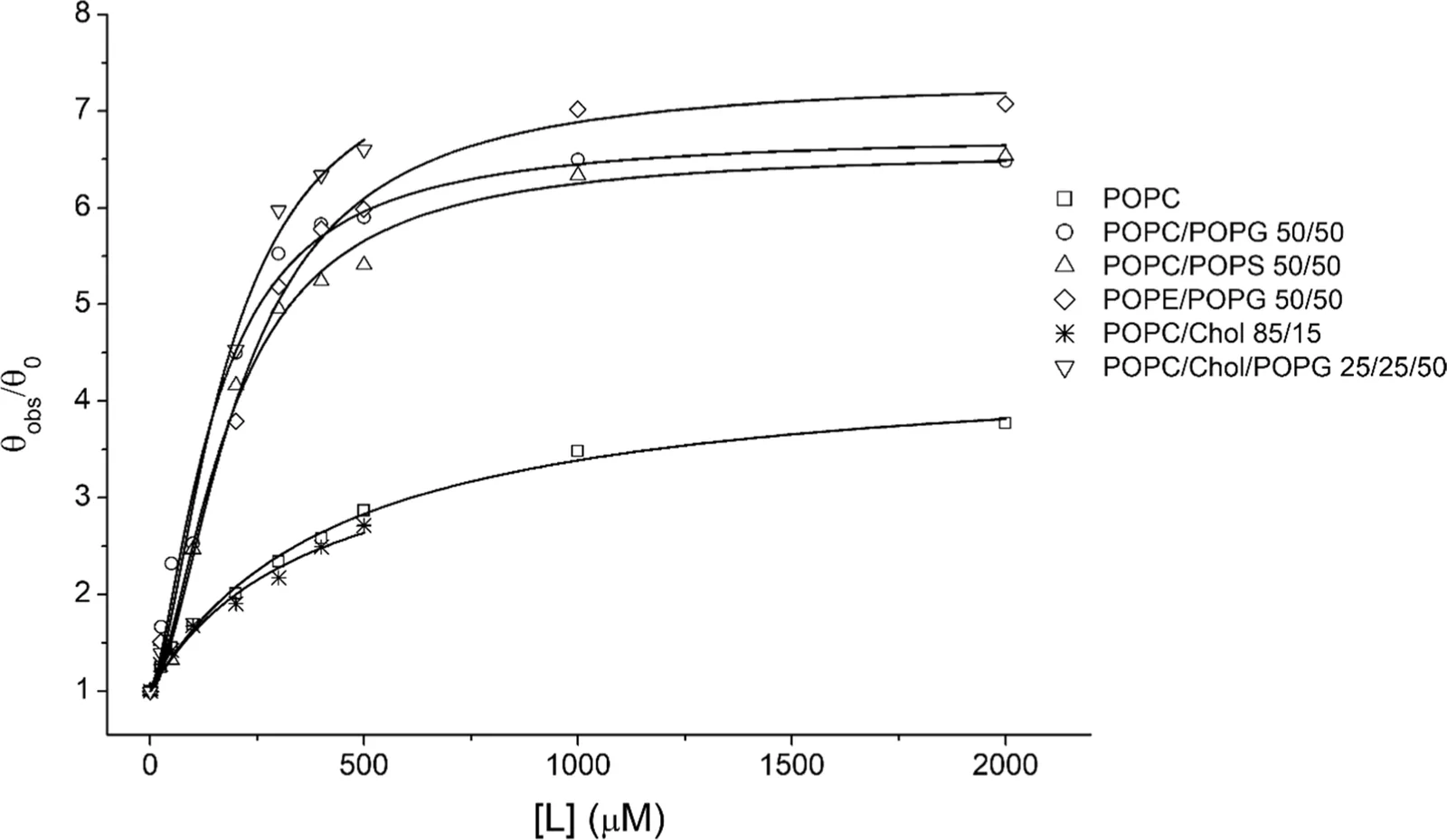
CD-derived membrane-association isotherms of Ctx­(Ile^21^)-Ha in lipid vesicles of different compositions. The normalized mean residue ellipticity at 222 nm, θ_obs_/θ_0_, is plotted as a function of total lipid concentration, [*L*]. Solid lines represent nonlinear fits to the coupled membrane binding/folding model described by eq 2.

**Table 1:** CD-Derived Conformational and Membrane-Association Parameters of Ctx­(Ile^21^)-Ha in Liposomes of Different Compositions^a^ ^,^ ^b^

System	θ_222_ (deg·cm^2^·dmol^–1^)	θ_b_ (deg·cm^2^·dmol^–1^)	*f*_b_ at [*L*]_max_	Estimated α-helical content (%)	*K*_app_ × 10^3^ (M^–1^)
POPC	–13,519	–15,876	0.81	50.4	4.6 ± 0.3
POPC/Chol 85/15	–7659	–10,634	0.62	34.7	5 ± 2
POPC/Chol 75/25	–5870	n.d.	n.d.	20.3*	n.d.
POPC/Chol 60/40	–4709	n.d.	n.d.	16.8*	n.d.
POPC/Erg 75/25	–6489	n.d.	n.d.	22.2*	n.d.
POPC/POPG 50/50	–23,245	–24,233	0.95	75.6	13 ± 1 (*n*_H_ = 1.5 ± 0.2)
POPC/POPS 50/50	–23,436	–23,666	0.99	73.9	11 ± 2 (*n*_H_ = 1.6 ± 0.2)
POPE/POPG 50/50	–25,354	–26,306	0.96	81.8	9.4 ± 0.9 (*n*_H_ = 1.7 ± 0.3)
POPC/Chol/POPG 25/25/50	–18,312	–21,571	0.82	67.6	11 ± 2 (*n*_H_ = 1.6 ± 0.4)

a θ_222_ corresponds to the experimental mean residue ellipticity at 222 nm at the highest lipid concentration tested ([*L*]_max_): 2 mM for POPC, POPC/POPG 50/50, POPC/POPS 50/50, and POPE/POPG 50/50, and 0.5 mM for the remaining systems. θ_b_ represents the limiting ellipticity of the membrane-associated state obtained from the binding/folding fit. The membrane-associated fraction at [*L*]_max_, *f*_b_, was calculated from the population-weighted ellipticity relationship using the experimentally determined θ_0_ from the corresponding titration series. When θ_b_ could be reliably determined, the α-helical content of the limiting membrane-associated state was calculated from θ_b_ using eq 1; otherwise, the α-helical content was estimated from θ_222_ at [*L*]_max_ and represents an apparent ensemble-averaged value (*). θ_b_, *K*_app_, and *n*_H_ were obtained from the fit using eq 2. Hill coefficients (*n*_H_) are shown in parentheses together with the corresponding lipid composition. Ctx­(Ile^21^)-Ha concentration was 30 μM, and measurements were performed at 25 °C in 10 mM potassium phosphate buffer (pH 7.0).

b n.d., not determined.

POPC induced a progressive increase in the α-helical CD signature (Figure 2A). At lower POPC concentrations, the spectra reflect population-weighted contributions from predominantly disordered peptide in solution and membrane-associated peptide adopting α-helical structure. Although the experimentally accessible POPC concentrations did not fully reach the limiting CD response, the nonlinear analysis of the titration yielded *K*_app_ = (4.6 ± 0.3) × 10^3^ M^–1^ and θ_b_ = −15,876 deg·cm^2^·dmol^–1^. The latter corresponds to an estimated α-helical content of 50.4% for the limiting membrane-associated state. At 2 mM POPC, approximately 81% of the CD-monitored transition was reached (Table 1).

Cholesterol progressively attenuated the membrane-induced conformational response (Figure 2B–D). At 15 mol % cholesterol, the spectra still developed α-helical features, particularly at the highest lipid concentrations, although the conformational response progressed more slowly than in POPC. This allowed the titration to be analyzed using the same coupled membrane binding/folding model, yielding *K*_app_ = (5 ± 2) × 10^3^ M^–1^, which is indistinguishable within the experimental uncertainty from the value obtained for POPC. In contrast, the limiting ellipticity was substantially less negative (−10,634 deg·cm^2^·dmol^–1^), corresponding to an α-helical content of only 34.7%. Thus, at 15 mol % cholesterol, the main effect is not a measurable reduction in the apparent membrane-association parameter, but rather a marked limitation of helix stabilization in the membrane-associated state. At 25 and 40 mol % cholesterol, the conformational response was further attenuated, and no limiting state could be reliably defined. The apparent ensemble-averaged α-helical contents at the highest lipid concentration tested were 20.3 and 16.8%, respectively. Importantly, the inability to determine *K*_app_ under these conditions does not imply the absence of membrane association. Peptide interaction with cholesterol-containing vesicles is independently supported by the changes in zeta potential reported in Table S1 and by the monolayer experiments, although the latter showed reduced peptide penetration relative to POPC. Rather, the CD-monitored conformational transition was insufficiently developed to support reliable fitting. This progressive attenuation is consistent with the well-established role of cholesterol in increasing bilayer packing density and mechanical rigidity, thereby limiting helix stabilization and, at higher cholesterol contents, reducing the overall membrane-induced conformational response.^56^

Ergosterol produced a similarly limited conformational response (Figure 2E). POPC/Erg (75/25 mol/mol) yielded only partial α-helical features, corresponding to an apparent ensemble-averaged α-helical content of 22.2% at the highest lipid concentration tested, and no reliable limiting state could be obtained from the titration. Although ergosterol displays characteristic CD absorption features in the near-UV region,^57,58^ these features were not detectably altered by Ctx­(Ile^21^)-Ha (Figure S2), indicating no measurable peptide-induced perturbation of the ergosterol environment under our experimental conditions. Together with the cholesterol results, these findings are consistent with sterols acting primarily as membrane-ordering modulators that attenuate the membrane-induced conformational response of Ctx­(Ile^21^)-Ha.

In contrast, anionic membranes strongly promoted both membrane association and α-helical folding (Figures 2F–I and 3). POPC/POPG 50/50, POPC/POPS 50/50, and POPE/POPG 50/50 yielded *K*_app_ values of 13 ± 1, 11 ± 2, and 9.4 ± 0.9 × 10^3^ M^–1^, respectively, approximately 2- to 3-fold higher than that obtained for POPC. Their limiting values corresponded to α-helical contents of 75.6, 73.9, and 81.8%, respectively. The respective titrations reached 95–99% of their limiting CD responses at 2 mM lipid, supporting the robustness of the derived membrane-associated conformations. Despite the different monolayer responses observed for PG- and PS-containing systems, POPC/POPG and POPC/POPS therefore produced essentially comparable limiting α-helical contents. The anionic systems also displayed Hill coefficients greater than unity (*n*_H_ ∼ 1.5–1.7), which indicate an apparent positive cooperativity in the CD-monitored membrane binding/folding response. These trends are also consistent with the zeta-potential measurements, which showed shifts toward less negative values upon peptide addition to anionic vesicles (Table S1), supporting peptide accumulation at the membrane interface.

The ternary POPC/Chol/POPG (25/25/50) system further illustrates the interplay between membrane charge and sterol-induced packing. Despite the presence of 25 mol % cholesterol, the peptide retained a relatively high *K*_app_ of (11 ± 2) × 10^3^ M^–1^ (*n*_H_ = 1.6 ± 0.4), comparable to the other anionic systems. However, its limiting θ_b_ of −21,571 deg·cm^2^·dmol^–1^ corresponded to 67.6% α-helical structure, lower than that observed for POPC/POPG in the absence of cholesterol. Thus, the anionic component largely preserves the favorable membrane-association response, whereas cholesterol attenuates the extent of helix stabilization within the membrane-associated state.

Overall, the CD analysis demonstrates that anionic membranes favor both the coupled membrane-association response and stabilization of the α-helical state of Ctx­(Ile^21^)-Ha, whereas sterol-containing zwitterionic membranes markedly attenuate membrane-induced folding. These trends are consistent with the monolayer and zeta-potential measurements, which independently support peptide interaction with the membrane interface, together with enhanced penetration into anionic lipid films and reduced penetration in sterol-containing monolayers. Importantly, the results also show that membrane association and helix stabilization are related but not equivalent observables, as illustrated by POPC and POPC/Chol (85/15), which display comparable values but substantially different limiting α-helical contents. These findings are consistent with the monolayer experiments, which showed greater peptide penetration into anionic lipid films and reduced penetration in sterol-containing monolayers, supporting a close relationship between favorable membrane interaction and helix stabilization for Ctx­(Ile^21^)-Ha.

### Membrane Charge and Packing Dictate the Local Dynamics of TOAC-Labeled Ctx­(Ile^21^)-Ha Analogues

To directly compare the backbone dynamics of Ctx­(Ile^21^)-Ha in different lipid environments, the peptide was site-specifically labeled with the paramagnetic amino acid TOAC at positions 2 and 13, generating the analogues [TOAC^2^]­Ctx­(Ile^21^)-Ha and [TOAC^13^]­Ctx­(Ile^21^)-Ha. These analogues were subsequently investigated by continuous-wave ESR to probe local peptide dynamics, motional restriction, and membrane-induced ordering at distinct regions of the peptide backbone, following the well-established application of site-directed spin labeling ESR to investigate lipid–protein and peptide–membrane interactions.^59^ ESR spectra of [TOAC^2^]­Ctx­(Ile^21^)-Ha and [TOAC^13^]­Ctx­(Ile^21^)-Ha were acquired in the presence of POPC, POPC/Chol, POPC/Erg, POPC/POPG, and POPE/POPG liposomes over a wide range of lipid concentrations. Overall, both TOAC-labeled analogues displayed sharp, narrow ESR spectra in aqueous solution, consistent with high rotational mobility of the TOAC spin label and the peptide segment to which it is attached. Upon LUV titration, the spectra became progressively broadened, reflecting reduced local mobility of the spin label due to peptide association with the lipid membranes (Figures 4 and S3, S4).^44,45^

**Figure 4:**
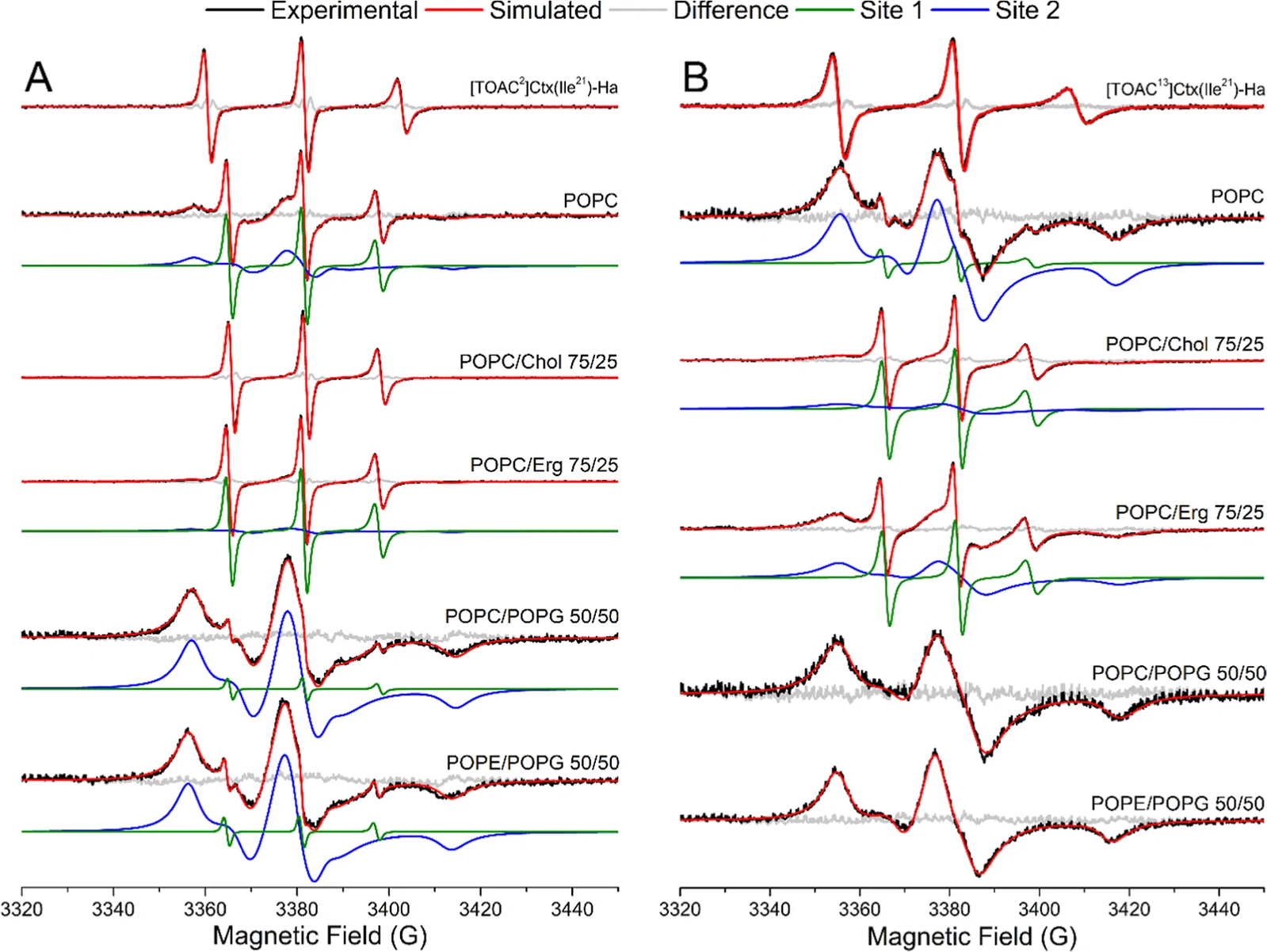
NLLS spectral simulations of (A) [TOAC^2^]­Ctx­(Ile^21^)-Ha and (B) [TOAC^13^]­Ctx­(Ile^21^)-Ha in the presence of 1 mM liposomes. Experimental (black) and best-fit simulated (red) spectra are illustrated along with the mobile (green) and ordered membrane-associated (blue) components. The difference between experimental and simulated spectra is shown in gray. Peptide concentration was 50 μM for POPC and 100 μM for the remaining systems. Measurements were performed in 20 mM potassium phosphate buffer (pH 7.0) at 25 °C.

Notably, the two analogues displayed distinct spectral line shapes even in solution. [TOAC^2^]­Ctx­(Ile^21^)-Ha exhibited sharper spectral features than [TOAC^13^]­Ctx­(Ile^21^)-Ha, reflecting the higher conformational freedom expected from a probe located near the N-terminus. In contrast, the TOAC residue positioned closer to the center of the peptide sequence experiences stronger steric and backbone constraints and is also consistent with the higher α-helical propensity previously reported for [TOAC^13^]­Ctx­(Ile^21^)-Ha in solution.^45,60,61^ Accordingly, nonlinear least-squares (NLLS) spectral simulations indicated that the rotational diffusion (*R̅*) of the spin probe in [TOAC^2^]­Ctx­(Ile^21^)-Ha peptide is approximately 2-fold higher than that observed for [TOAC^13^]­Ctx­(Ile^21^)-Ha (*R̅* = 5.13 × 10^8^ s^–1^ versus 2.45 × 10^8^ s^–1^, Table 2). Additionally, in [TOAC^2^]­Ctx­(Ile^21^)-Ha, the spin label replaces the native tryptophan residue, which can act as an interfacial anchor in peptide–membrane systems and also contribute to membrane insertion and pore-forming behavior in membrane-active peptides.^62–65^ This substitution may therefore weaken hydrophobic and interfacial interactions that would otherwise stabilize peptide–membrane contacts.

**Table 2:** Dynamic and Structural Parameters Obtained from NLLS Spectral Simulations of ESR Spectra of TOAC-Labeled Ctx­(Ile^21^)-Ha^a^ ^,^ ^b^

	[TOAC^2^]Ctx(Ile^21^)-Ha					[TOAC^13^]Ctx(Ile^21^)-Ha				
	*R̅* (10^7^ s^–1^)		*S*_0_	Population (%)		*R̅* (10^7^ s^–1^)		*S*_0_	Population (%)	
System	site 1	site 2	site 2	site 1	site 2	site 1	site 2	site 2	site 1	site 2
buffer	51.3	-	-	100	0	24.5	-	-	100	0
POPC	55.0	0.347	0.67	5	95	25.7	0.219	0.72	1	99
POPC/Chol	55.0	-	-	100	0	25.1	0.162	0.72	18	82
POPC/Erg	52.5	0.224	0.67	51	49	26.3	0.155	0.75	7	93
POPC/POPG	55.0	0.347	0.68	3	97	-	0.102	0.75	0	100
POPE/POPG	55.0	0.692	0.64	3	97	-	0.115	0.73	0	100

a Spectra of the peptides in buffer and in the presence of 1 mM POPC LUVs were acquired at 50 μM peptide. Spectra in the presence of 1 mM POPC/POPG (50/50 mol/mol), POPE/POPG (50/50 mol/mol), POPC/Chol (75/25 mol/mol), and POPC/Erg (75/25 mol/mol) were acquired at 100 μM peptide. Site 1 and site 2 correspond to the mobile and membrane-associated/ordered spectral components, respectively. Reported parameters include the average rotational diffusion *R̅*, order parameter *S*_0_, and the relative population of each spectral component.

b (-) indicates that the spectral component could not be fitted.

Upon lipid titration, a second and progressively broadened spectral component emerged in the ESR spectra of both TOAC-labeled analogues (Figures 4 and S3, S4), indicating peptide association with the lipid bilayers accompanied by reduced local backbone mobility. Indeed, for most membrane conditions, the experimental spectra could only be satisfactorily fitted using a two-component model. NLLS simulations indicate that the narrow component retained the high mobility characteristic of the free peptide in solution, whereas the broadened component exhibited a substantially lower *R̅* (2.24–6.92 × 10^6^ s^–1^ for [TOAC^2^]­Ctx­(Ile^21^)-Ha and 1.02–2.19 × 10^6^ s^–1^ for [TOAC^13^]­Ctx­(Ile^21^)-Ha) together with higher order parameter (*S*_0_) values (0.64–0.68 for [TOAC^2^]­Ctx­(Ile^21^)-Ha and 0.72–0.75 for [TOAC^13^]­Ctx­(Ile^21^)-Ha), consistent with membrane association and motional restriction. Although slight differences in TOAC rotational diffusion within the membrane-associated state were observed depending on lipid composition, the order parameter values for each peptide remained remarkably similar across all membrane systems (Table 2). This result suggests that once the peptide becomes membrane-bound and structurally organized, the local ordering sensed by the spin label is largely independent of membrane composition, whereas the residual backbone mobility remains moderately sensitive to lipid environment. Notably, the TOAC probe positioned at the N-terminus consistently displayed higher mobility and lower order than the probe located closer to the central region of the peptide, putatively within the helical segment, indicating greater conformational freedom of the N-terminal region upon membrane interaction.

The relative population of the broadened membrane-associated component was also strongly dependent on membrane composition. In general, this ordered component became substantially more pronounced in sterol-free membranes (95–100%, Table 2), indicating stronger peptide conformational restriction under these conditions. Notably, [TOAC^13^]­Ctx­(Ile^21^)-Ha consistently displayed larger populations of the membrane-associated component than [TOAC^2^]­Ctx­(Ile^21^)-Ha under the same experimental conditions, further supporting the stronger restriction experienced by the central region of the peptide upon membrane binding. In contrast, sterol-containing membranes exhibited considerably larger populations of the mobile component (51–100% for [TOAC^2^]­Ctx­(Ile^21^)-Ha and 7–18% for [TOAC^13^]­Ctx­(Ile^21^)-Ha), corresponding to free peptide in solution, suggesting that cholesterol and ergosterol partially hinder peptide partitioning into the bilayer. Interestingly, cholesterol reduced the fraction of membrane-bound peptide more efficiently than ergosterol, particularly for [TOAC^2^]­Ctx­(Ile^21^)-Ha. In fact, no broadened ordered component could be detected for this peptide analogue in POPC/Chol membranes. These findings indicate either distinct peptide affinities toward cholesterol- and ergosterol-containing membranes or, alternatively, that the N-terminal segment remains comparatively flexible and less sensitive to sterol-induced membrane packing constraints.

Overall, the ESR results reveal a strong membrane-dependent restriction of peptide dynamics, with POPC and anionic bilayers producing predominantly motionally restricted spectral populations, whereas sterol-containing membranes retain larger mobile fractions, particularly for [TOAC^2^]­Ctx­(Ile^21^)-Ha. These findings are qualitatively consistent with the CD and monolayer experiments, which independently showed favorable membrane association and helix stabilization in anionic systems and reduced peptide penetration and conformational response in sterol-containing membranes.

## Conclusions

Ctx­(Ile^21^)-Ha behaves as a membranotropic peptide whose interaction with lipid membranes is governed by the interplay between membrane surface charge and lipid packing. Langmuir monolayers showed that anionic lipid identity and mole fraction modulate peptide penetration and the resulting perturbation of interfacial lipid packing, whereas sterols restrict peptide expansion and attenuate peptide-induced membrane perturbation. CD further revealed that negatively charged bilayers strongly favor membrane association coupled to α-helical stabilization. POPC/POPG and POPC/POPS produced comparable limiting α-helical contents, despite their distinct responses in the monolayer experiments, indicating that anionic headgroup chemistry can modulate interfacial penetration without necessarily producing corresponding differences in the final membrane-associated secondary structure.

ESR experiments further showed that the central region of the peptide, likely associated with the helical segment, becomes substantially more immobilized upon membrane interaction than does the N-terminal region. ESR also revealed dynamic heterogeneity with coexistence of mobile and motionally restricted peptide populations. POPC and anionic bilayers produced predominantly motionally restricted populations, whereas sterol-containing membranes retained larger mobile fractions, particularly for [TOAC^2^]­Ctx­(Ile^21^)-Ha, highlighting the sensitivity of peptide dynamics to membrane packing. In parallel, the CD-derived membrane-association analysis and ζ-potential measurements showed that the membrane association can persist even when sterols attenuate helix stabilization and monolayer penetration, reinforcing that membrane association, peptide folding, and membrane penetration are mechanistically related but distinct steps in AMP membrane activity.

Overall, the interaction of Ctx­(Ile^21^)-Ha with biomimetic membranes is best described as a charge- and packing-modulated process, in which electrostatic interactions favor peptide recruitment and folding, whereas membrane order modulates the extent of membrane penetration. These findings provide a detailed biophysical framework for understanding the membranotropic behavior and membrane selectivity of Ctx­(Ile^21^)-Ha in biologically relevant membrane environments.

## Materials and Methods

### Synthesis, Purification, and Characterization of Ctx­(Ile^21^)-Ha and TOAC-Labeled Analogues

Ctx­(Ile^21^)-Ha (GWLDVAKKIGKAAFNVAKNFI-NH_2_, molecular weight, 2289.75 g mol^–1^; purity, 96%) and its two paramagnetic TOAC-labeled analogues [TOAC^2^]­Ctx­(Ile^21^)-Ha (G**O**LDVAKKIGKAAFNVAKNFI-NH_2_, molecular weight, 2299.75 g mol^–1^; purity, 97%) and [TOAC^13^]­Ctx­(Ile^21^)-Ha (GWLDVAKKIGKA**O**FNVAKNFI-NH_2_, molecular weight, 2414.9 g mol^–1^; purity, 96%), where “**O**” represents the spin-labeled amino acid TOAC (2,2,6,6-tetramethylpiperidine-*N*-oxyl-4-amino-4-carboxylic acid), were synthesized by solid-phase peptide synthesis. Detailed procedures regarding peptide synthesis, purification, physicochemical characterization, and determination of peptide properties have been previously described by Vicente et al.^45^

### Adsorption and Insertion into Lipid Monolayers

Peptide adsorption at the air–water interface and insertion into preformed lipid monolayers were investigated using a custom-built circular Langmuir trough (surface area of 7 cm^2^ and subphase volume of 4.5 mL) under constant-area conditions.

Adsorption assays were carried out by adding increasing volumes of peptide diluted in saline solution (20 mM potassium phosphate buffer, pH 7.0, containing 150 mM NaCl) into the aqueous subphase under continuous stirring. The resulting variation in surface pressure (Δπ) was monitored over time until equilibrium was reached.

Surface activity profiles (Δπ as a function of peptide concentration, *c*) were analyzed using nonlinear least-squares fitting, following the procedures detailed in Alvares et al.^54^ The adsorption isotherms were then used to determine the minimum peptide concentration capable of inducing the maximum surface pressure variation, and this concentration was subsequently employed in monolayer insertion experiments.

To investigate peptide insertion into lipid monolayers, phospholipids dissolved in a chloroform/methanol solution (2:1 v/v) were carefully spread onto the air–saline interface until reaching an initial surface pressure of 30 mN/m, a value comparable to the lipid density found in cell membranes. Following monolayer formation, the peptide was injected into the subphase at a final concentration of 0.7 μM under continuous stirring. Peptide insertion into the monolayers was monitored by recording the surface pressure increase over time. All experiments were conducted at 24 °C. The standard deviations were obtained from at least three independent measurements.

### Large Unilamellar Vesicles (LUVs) Preparation

The lipids 1-palmitoyl-2-oleoyl-*sn*-glycero-3-phosphocholine (POPC), 1-palmitoyl-2-oleoyl-*sn*-glycero-3-phosphoethanolamine (POPE), 1-palmitoyl-2-oleoyl-*sn*-glycero-3-phospho-(1′-rac-glycerol) (POPG), 1-palmitoyl-2-oleoyl-*sn*-glycero-3-phospho-l-serine (POPS), ergosterol (Erg), and cholesterol (Chol) were purchased from Avanti Polar Lipids, Inc.

LUVs were prepared as described elsewhere.^66^ Briefly, appropriate aliquots of lipid stock solutions in chloroform were mixed at the desired molar ratios in a glass tube. The organic solvent was evaporated under a gentle N_2_ flow until a thin lipid layer was formed. The lipid films were then dried for 2 h under vacuum to remove residual solvent traces. Hydration was performed with 10 mM potassium phosphate buffer (pH 7.0) to promote multilamellar vesicle (MLV) formation. The resulting MLVs were subjected to six freeze–thaw cycles, ensuring a homogeneous dispersion, and then extruded 40 times using an Avanti mini-extruder (Avanti Polar Lipids, Inc.) to obtain a homogeneous LUV dispersion. Extrusion was performed through a 100 nm polycarbonate filter at a temperature kept at least 10 °C above the lipid mixture phase transition temperature.

To mimic distinct biological membrane environments, we employed a set of lipid mixtures that resembles the defining traits of distinct cellular membranes, such as POPC, as a simplified model of mammalian bilayers; POPC/Chol (75/25 mol/mol), to retain the ordering effect of cholesterol in human membranes;^67,68^ POPC/Erg (75/25 mol/mol), to incorporate the main sterol found in fungal membranes;^69–71^ POPC/POPG (50/50 mol/mol), as a simplified model of Gram-positive bacterial membranes;^29,30^ and POPE/POPG (50/50 mol/mol), representing selected features of Gram-negative bacterial membranes.^29,72^ The 50 mol % anionic lipid content was selected as a simplified comparative condition to provide a robust spectroscopic response and to enable direct comparison of different anionic headgroups at the same nominal anionic lipid fraction. Additionally, POPC/Chol (60/40 and 85/15 mol/mol), POPC/Chol/POPG (25/25/50 mol/mol/mol), and POPC/POPS (50/50 mol/mol) were used to investigate the influence of cholesterol content and anionic headgroups with distinct chemical structures, such as phosphatidylserine compared to phosphatidylglycerol.

### Circular Dichroism Spectroscopy

CD experiments were performed on a Jasco J-815 spectropolarimeter equipped with a Peltier temperature control system. Spectra were recorded in a 1 mm path-length quartz cuvette at 25 °C using a scan speed of 50 nm/min over the wavelength range from 270 to 190 nm. For ergosterol-containing samples, spectra were collected from 350 to 190 nm to account for sterol-associated spectral contributions. Data pitch and bandwidth were set to 1 and 2 nm, respectively. Peptide stock solutions (0.35 mM in ultrapure water) were diluted in 10 mM potassium phosphate buffer (pH 7.0) to a final concentration of 30 μM. CD spectra were recorded in the absence and presence of LUVs for all lipid compositions investigated. Six consecutive scans were recorded for each sample, and the best-quality scans were averaged. The corresponding blanks (buffer or LUV suspensions) were subtracted from the spectra, which were subsequently baseline-corrected and converted to MRE, expressed in deg·cm^2^·dmol^–1^. Data processing was carried out using the CDToolX software.^73^

The α-helical fraction (*f*_H_) was estimated from the MRE at 222 nm according to the method derived from Luo and Baldwin^55,66^

1 $$f_{H}=\frac{\theta_{222}-\theta_{C}}{\theta_{H}-\theta_{C}}$$

where θ_C_ = 2220 – 53*T* and $\theta_{H}=\left(250T-44,000\right)\left(1-\frac{3}{N}\right)$ . Here, θ_222_ is the experimental MRE at 222 nm, whereas θ_C_ and θ_H_ are the limiting MRE values for fully disordered and fully α-helical conformations, respectively. *T* is the experimental temperature in degrees Celsius, and *N* is the number of amino acid residues in the peptide.

Membrane association was analyzed assuming a coupled membrane binding/folding model, in which the lipid-dependent change in ellipticity at 222 nm reports the transition from the predominantly disordered peptide in solution toward the membrane-associated conformational state.^58^ Binding isotherms were constructed by plotting the normalized molar ellipticity, θ_obs_/θ_0_, as a function of lipid concentration [*L*], where (θ_obs_) and absence (θ_0_) are the MRE values at 222 nm in the presence and absence of LUVs, respectively. The binding equation previously used by Crusca et al.^66^ was adapted to a Hill formalism to account for the cooperative CD response observed for some anionic membrane systems

2 $$\frac{\theta_{obs}}{\theta_{0}}=1+\left(\frac{\theta_{b}}{\theta_{0}}-1\right)\left(\frac{{\left(\frac{K_{app}\left[L\right]}{2}\right)}^{n_{H}}}{1+{\left(\frac{K_{app}\left[L\right]}{2}\right)}^{n_{H}}}\right)$$

Here, θ_b_ is the limiting MRE at 222 nm corresponding to the membrane-associated state, *K*_app_ is the apparent membrane-association parameter obtained from the CD-monitored binding/folding transition, *n*_H_ is the Hill coefficient, and [*L*]/2 represents the effective lipid concentration accessible for peptide association, assuming that binding occurs predominantly at the outer leaflet of the LUVs. For *n*_H_ = 1, the equation reduces to a noncooperative binding response. For systems reaching a well-defined titration plateau, the limiting θ_b_ value was used to estimate the α-helical content. For POPC, θ_b_ was obtained from the nonlinear fit of the binding isotherm. For sterol-containing systems without a limiting plateau, the [θ]_222_ value at the highest lipid concentration tested was used, yielding an apparent ensemble-averaged α-helical content.

### Electron Spin Resonance Spectroscopy and Nonlinear Least-Squares Spectral Simulations

Continuous-wave ESR spectroscopy was performed as previously described by Vicente et al.^45^ ESR spectra were acquired at 25 °C using a Varian E109 spectrometer operating at 9.5 GHz, employing a microwave power of 5 mW and modulation amplitudes ranging from 0.5 to 1.5 G. Peptide–lipid mixtures were prepared in 20 mM potassium phosphate buffer (pH 7.0) containing 150 mM NaCl using peptide concentrations ranging from 50 to 100 μM. For POPC titrations, 50 μM peptide was used to extend the accessible *L*/*P* range while maintaining an adequate ESR signal-to-noise ratio; 100 μM peptide was used for the remaining membrane systems. Samples were loaded into quartz capillaries at peptide-to-lipid molar ratios of 2:1, 1:1, 1:2, 1:4, 1:5, 1:6, 1:8, 1:10, and 1:50.

Spectral simulations were carried out using the LabVIEW-based (National Instruments) “Multicomponent” software developed by Christian Altenbach (University of California, Los Angeles, CA).^74^ Initial values for the *g* and hyperfine tensors were taken from previous studies^45^ and allowed to vary slightly during the fitting process. The final set of the *g* tensor components used in the simulations were *g*_*xx*_ = 2.0090, *g*_*yy*_ = 2.0060, and *g*_*zz*_ = 2.0030. Hyperfine tensor values were set to *A*_*xx*_ = 7.1 G and *A*_*yy*_ = 5.8 G for the ordered component, and *A*_*xx*_ = *A*_*yy*_ = 6.0 G for the mobile site. The dynamic and structural parameters, including the average rotational diffusion tensor *R̅*, correlation time τ, and order parameter *S*_0_ were obtained from the NLLS simulations as described elsewhere.^45,75^ The best-fit values for *A*_*zz*_, *R̅*, τ, and *S*_0_ are summarized in Tables S2 and S3.

### Dynamic Light Scattering and Zeta Potential (ζ) Measurements

The hydrodynamic diameter of the LUVs was determined by dynamic light scattering (DLS) using a Litesizer 500 analyzer (Anton Paar, Graz, Austria), which was also used for zeta potential measurements. For DLS analysis, the refractive indices of the buffer solution (10 mM potassium phosphate, pH 7.0) and lipid phase were set to 1.33 and 1.45, respectively, using the built-in Kalliope software library. Transmittance values varied between 75 and 85% for all measurements. Instrument parameters including laser power adjustment, quality mode, filter mode, focus mode, and scattering angle were automatically optimized by the Kalliope software. DLS measurements were performed using disposable low-volume cuvettes (Eppendorf, Inc.). ζ measurements of liposomes with or without peptide were carried out using disposable Omega capillary cuvettes, and ζ potential values were calculated according to the Smoluchowski approximation model.^76^ The lipid and peptide concentrations were fixed at 100 and 2 μM, respectively, yielding a final lipid-to-peptide molar ratio of 50. All measurements were performed at 25 °C in triplicate.

## Supplementary Material



## Notes

Far-UV CD spectrum of Ctx­(Ile^21^)-Ha in 75% (v/v) TFE, POPC/Erg CD control spectra, experimental ESR titration spectra for both TOAC-labeled analogues, DLS and zeta-potential characterization of LUVs, and NLLS spectral simulation parameters (PDF)
