# Supplementary material for: Membrane Charge and Packing Govern the Interfacial Insertion, Structure, and Dynamics of Ctx(Ile21)‑Ha and Its TOAC-Labeled Analogues in Biomimetic Liposomes
Source: ACS Omega. 2026 Oct 17;11(43):66980–91. doi: 10.1021/acsomega.6c07092 (PMC13737837; doi:10.1021/acsomega.6c07092)
Supplement: Supplementary file 1 [file ao6c07092_si_001.pdf]

Membrane charge and packing govern the interfacial insertion, structure and dynamics of Ctx(Ile^{21})-Ha and its TOAC-labeled analogues in biomimetic liposomes

Fadi S. S. Magalhães[||,a], Dayane S. Alvares[||,b], Rayssa P. R. Costa[c], Luis G. M. Basso[,a], and Eduardo F. Vicente[*,d].*

[||]*These authors contributed equally to this work.*

[*]*Shared corresponding authors.*

[a]Laboratory of Physical Sciences, Center of Sciences and Technology, State University of Northern Rio de Janeiro (UENF), Campos dos Goytacazes, RJ, Brazil.

[b]Department of Physics, Institute of Biosciences, Letters, and Exact Sciences, São Paulo State University (UNESP), São José do Rio Preto, SP, Brazil.

[c]Department of Animal Science, School of Agricultural and Veterinarian Sciences, São Paulo State University (UNESP), Jaboticabal, SP, Brazil.

[d]Department of Biosystems Engineering, School of Sciences and Engineering, São Paulo State University (UNESP), Tupã, SP, Brazil.

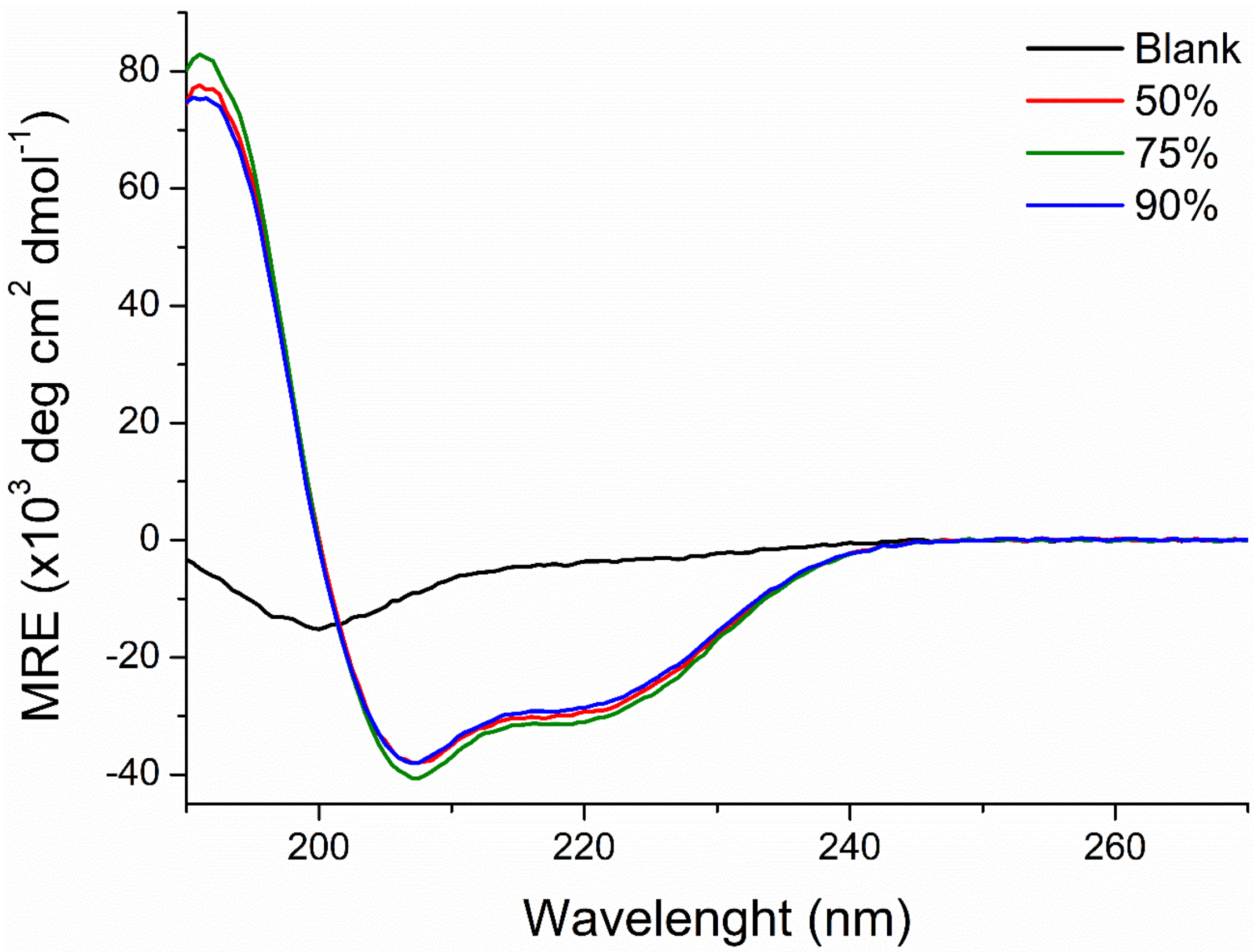

Figure S1. Far-UV CD spectra of 30 µM Ctx(Ile^{21})-Ha in the absence (blank) of 2,2,2-trifluoroethanol (TFE) and in the presence of 50, 75, and 90% (v/v) TFE. Spectra were recorded at 25 °C in TFE/10 mM potassium phosphate pH 7 mixtures and are expressed as mean residue ellipticity (MRE). Among the conditions tested, 75% (v/v) TFE produced the highest estimated α-helical content.

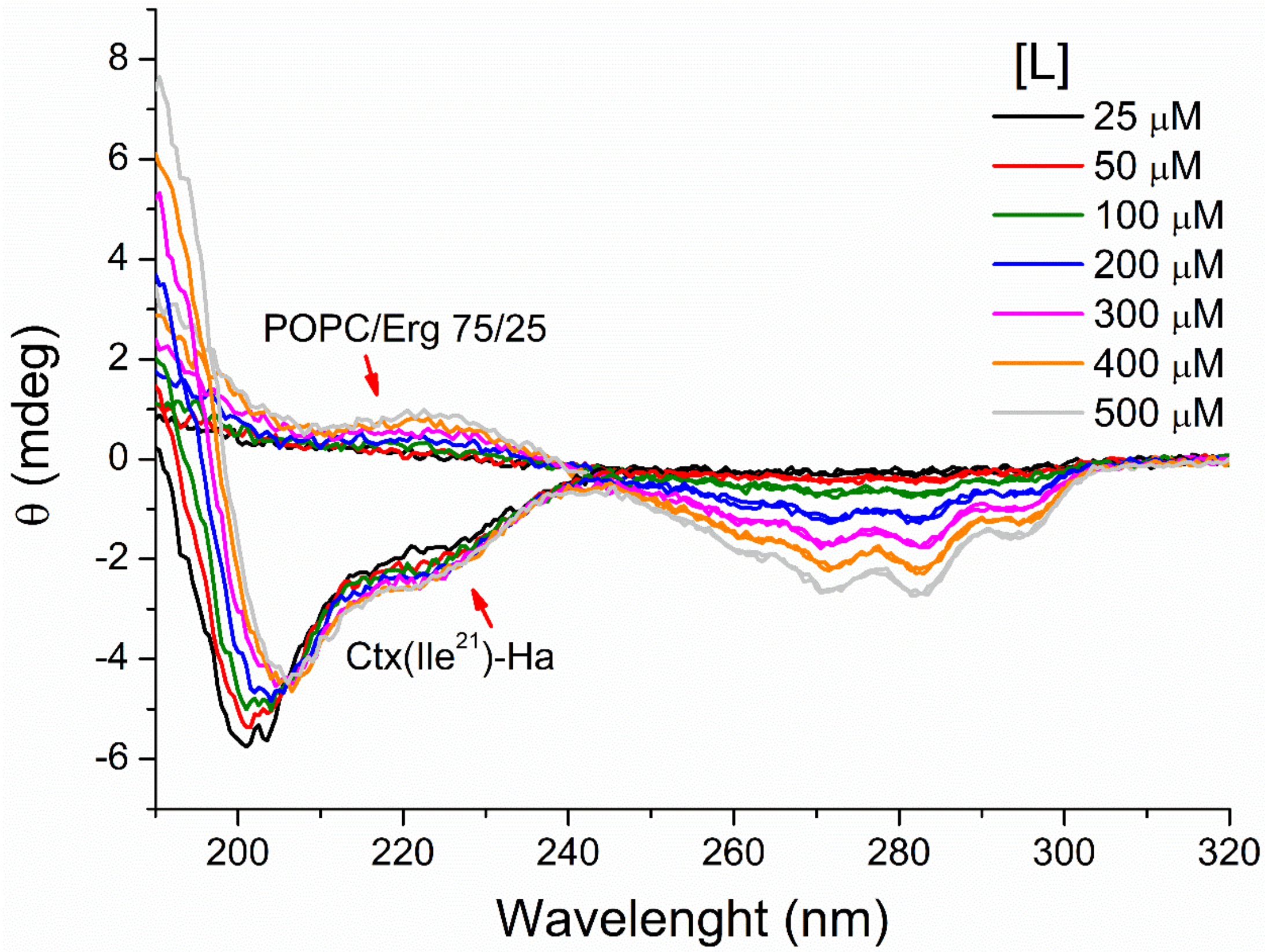

Figure S2. Far-UV CD spectra of 30 μM Ctx(Ile^{21})-Ha recorded in the presence of POPC/Erg (75/25 mol/mol) vesicles at increasing concentrations (25–500 μM) are shown alongside corresponding blank lipid controls. The blank spectra show characteristic CD signals from the ergosterol electronic transitions[1], and upon binding to Ctx(Ile^{21})-Ha no spectral alterations were detected in the ergosterol bands.

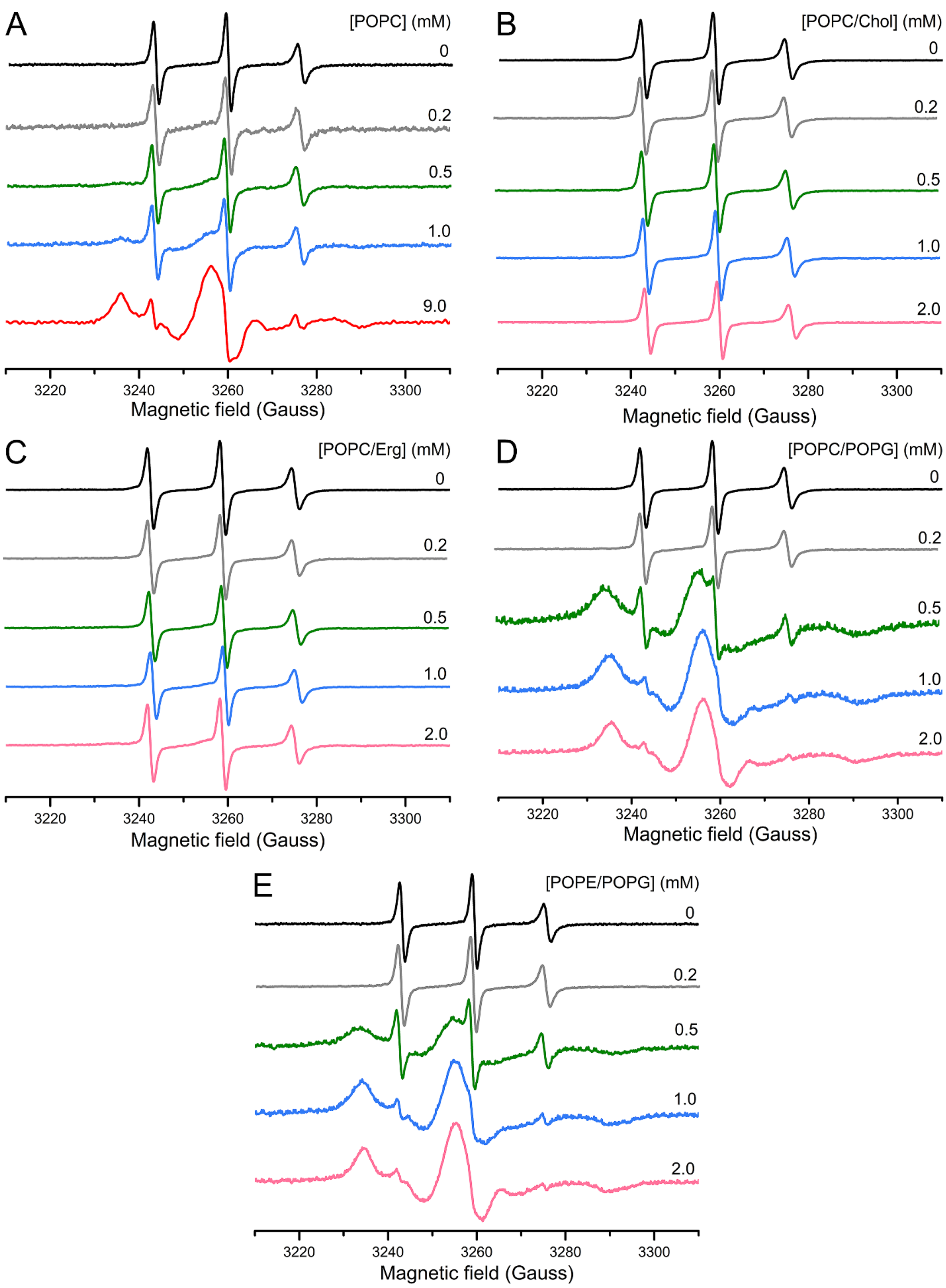

Figure S3. ESR spectra of [TOAC^2]-Ctx(Ile^{21})-Ha in the presence of increasing lipid concentrations of **(A)** POPC, **(B)** POPC/Chol (75/25 mol/mol), **(C)** POPC/Erg (75/25 mol/mol), **(D)** POPC/POPG (50/50 mol/mol), and **(E)** POPE/POPG (50/50 mol/mol). Progressive line broadening, where observed, reflects the appearance of a motionally restricted peptide population upon membrane association. No resolved restricted component was detected for [TOAC^2]Ctx(Ile^{21})-Ha in POPC/Chol membranes.

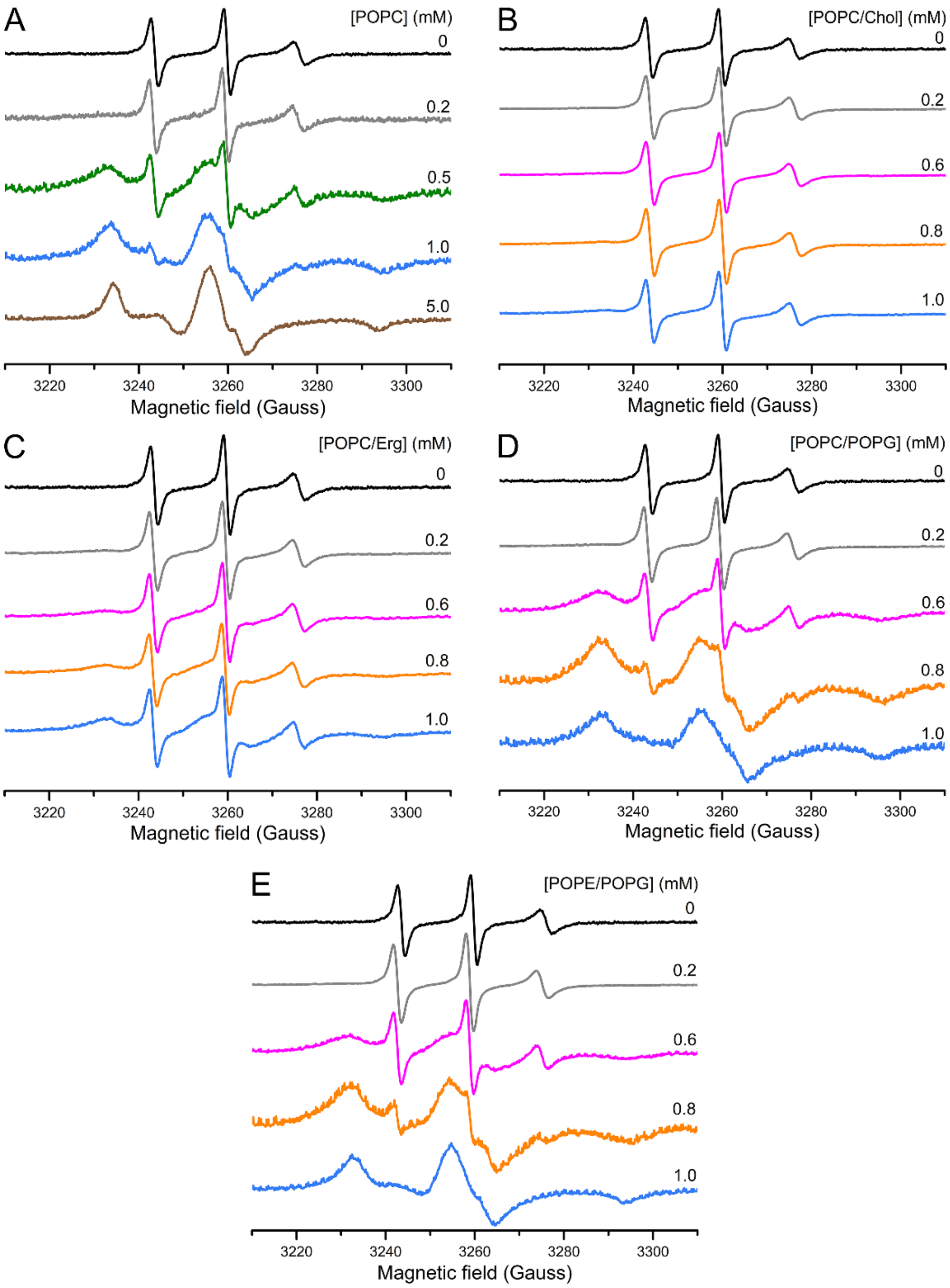

Figure S4. ESR spectra of [TOAC^{13}]-Ctx(Ile^{21})-Ha in the presence of increasing lipid concentrations of **(A)** POPC, **(B)** POPC/Chol (75/25 mol/mol), **(C)** POPC/Erg (75/25 mol/mol), **(D)** POPC/POPG (50/50 mol/mol), and **(E)** POPE/POPG (50/50 mol/mol). Progressive line broadening reflects peptide partitioning into the membranes and motional restriction upon binding.

Table S1. Dynamic light scattering (intensity-weighted hydrodynamic diameter, nm) and zeta potential values (mV) of lipid vesicles (100 μM) in the absence and presence of Ctx(Ile^{21})-Ha (2 μM).

**System**	**DLS (nm)**		**Zeta potential (mV)**	
	**Liposomes**	**+ Ctx(Ile^{21})-Ha**	**Liposomes**	**+ Ctx(Ile^{21})-Ha**
POPC	110 ± 23	128 ± 32	-3.0 ± 0.5	3.8 ± 0.6
POPC/Chol 75/25	116 ± 24	152 ± 49	-3.3 ± 0.6	0.2 ± 0.2
POPC/Chol 60/40	114 ± 25	161 ± 54	-3.0 ± 0.4	-1.7 ± 0.5
POPC/Chol 85/15	121 ± 33	138 ± 43	-2.0 ± 0.4	0.9 ± 0.4
POPC/Chol/POPG 25/25/50	116 ± 34	141 ± 49	-44 ± 4	-42 ± 2
POPC/Erg 75/25	102 ± 20	121 ± 33	-13 ± 1	-3.2 ± 0.4
POPC/POPG 50/50	119 ± 39	151 ± 47	-45 ± 2	-37 ± 2
POPC/POPS 50/50	113 ± 32	133 ± 45	-57 ± 3	-37 ± 2
POPE/POPG 50/50	135 ± 45	149 ± 53	-46 ± 4	-37 ± 2

Table S2. Dynamic and structural parameters obtained from NLLS spectral simulations of ESR spectra of [TOAC[2]]-Ctx(Ile[21])-Ha**.** Spectra of the peptide in buffer and POPC LUVs were acquired at 50 μM peptide, whereas in membrane-containing samples, spectra were acquired at 100 μM peptide. Site 1 and Site 2 correspond to the mobile and membrane-associated/ordered spectral components, respectively. Reported parameters include the hyperfine tensor A_{zz}, average rotational diffusion $\bar{R}$, order parameter S_0, correlation time τ, and the relative population of each spectral component. Spectra were acquired at 25 °C in potassium phosphate buffer, pH 7.0, containing 150 mM NaCl.

	A_{zz} (G)		$\bar{R}$ (10^8 s^{-1})		S_0	τ (ns)		Population (%)	
**[TOAC[2]]Ctx(Ile[21])-Ha (μM)**	Site 1	Site 2	Site 1	Site 2	Site 2	Site 1	Site 2	Site 1	Site 2
50	36.9		5.13			0.325			
**POPC/POPG 50/50 (μM)**									
100	36.9		5.37			0.310			
200	36.9	30.9	5.50	0.0708	0.62	0.303	23.5	41.6	58.4
500	37.0	32.0	5.50	0.0562	0.64	0.303	29.6	8.1	91.9
1000	37.0	32.0	5.50	0.0347	0.68	0.303	48.1	2.9	97.1
2000	37.0	32.0	5.62	0.0162	0.72	0.296	103	0.3	99.7
**POPE/POPG 50/50 (μM)**									
100	36.9		5.25			0.318			
200	36.9	32.3	5.25	0.204	0.53	0.318	8.16	38.4	61.6
500	36.9	32.4	5.37	0.123	0.58	0.310	13.5	7.3	92.7
1000	37.0	32.4	5.50	0.0692	0.64	0.303	24.1	2.6	97.4
2000	37.0	32.4	5.62	0.0457	0.69	0.296	36.5	0.2	99.8

**POPC (μM)**									
50	36.9		5.37			0.310			
100	36.9		5.37			0.310			
200	36.9		5.37	0.263	0.55	0.310	6.34		
500	36.9	30.5	5.50	0.0741	0.63	0.303	22.5	12.8	87.2
1000	36.9	30.5	5.50	0.0347	0.67	0.303	48.1	5.0	95.0
5000	36.9	30.9	5.50	0.0219	0.70	0.303	76.2	1.1	98.9
9000	36.9	30.9	5.50	0.0135	0.74	0.303	124	0.7	99.3
**POPC/Chol 75/25 (μM)**									
100	36.9		5.50			0.303			
200	36.9		5.50			0.303			
500	36.9		5.50			0.303			
1000	36.9		5.50			0.303			
2000	36.9		5.50			0.303			
**POPC/Erg 75/25 (μM)**									
100	36.9		5.13			0.325			
200	36.9		5.13			0.325			
500	36.9		5.25			0.318			
1000	36.9	30.1	5.25	0.022	0.67	0.318	74.4	51.2	48.9
2000	36.9	30.2	5.25	0.016	0.68	0.318	103	39.9	60.1

Table S3. Dynamic and structural parameters obtained from NLLS spectral simulations of ESR spectra of [TOAC[13]]-Ctx(Ile[21])-Ha**.** Spectra of the peptide in buffer and POPC LUVs were acquired at 50 μM peptide, whereas in membrane-containing samples, spectra were acquired at 100 μM peptide. Site 1 and Site 2 correspond to the mobile and membrane-associated/ordered spectral components, respectively. Reported parameters include the hyperfine tensor A_{zz}, average rotational diffusion $\bar{R}$, order parameter S_0, correlation time τ, and the relative population of each spectral component. Spectra were acquired at 25 °C in potassium phosphate buffer, pH 7.0, containing 150 mM NaCl.

	A_{zz} **(G)**		$\bar{R}$ **(10^8 s^{-1})**		S_0	τ **(ns)**		**Population (%)**	
**[TOAC[13]]Ctx(Ile[21])-Ha (μM)**	Site 1	Site 2	Site 1	Site 2	Site 2	Site 1	Site 2	Site 1	Site 2
50	36.8		2.45			0.679			
**POPC/POPG 50/50 (μM)**									
100	36.9		2.45			0.679			
200	36.9	32.2	2.45	0.0214	0.63	0.679	78.0	15.3	84.7
600	36.9	32.2	2.51	0.0170	0.68	0.664	98.1	2.2	97.8
800	37.4	32.2	2.57	0.0117	0.73	0.648	142	0.8	99.2
1000	38.3	32.2		0.0102	0.75		163	0	100
2000	38.3	32.2		0.00871	0.78		191	0	100
**POPE/POPG 50/50 (μM)**									
100	36.9		2.45			0.679			
200	36.9	31.5	2.45	0.0269	0.63	0.679	61.9	35.4	64.6
400	36.9	31.9	2.51	0.0219	0.64	0.664	76.2	9.2	90.8
600	36.9	32.4	2.63	0.0200	0.68	0.634	83.5	3.6	96.4
800	36.9	32.4	2.69	0.0132	0.73	0.619	126	1.4	98.6

1000		32.4		0.0115	0.73		145	0	100
**POPC (μM)**									
25	36.9		2.40			0.695			
50	37.0		2.40			0.695			
100	37.0		2.63			0.634			
200	37.0	32.0	2.57	0.0347	0.64	0.648	48.1	7.5	92.5
500	37.0	32.3	2.57	0.0263	0.70	0.648	63.4	2.1	97.9
1000	37.2	32.3	2.57	0.0219	0.72	0.648	76.2	0.85	99.15
5000		32.4		0.0174	0.75		95.9	0	100
**POPC/Chol 75/25 (μM)**									
100	36.8		2.45			0.679			
200	36.8		2.45			0.679			
400	36.9	33.3	2.45	0.0457	0.68	0.679	36.5	33.00	67.00
600	36.9	33.3	2.45	0.0427	0.69	0.679	39.1	22.00	78.00
800	36.9	32.2	2.45	0.0263	0.71	0.679	63.4	19.50	80.50
1000	36.9	32.4	2.51	0.0162	0.72	0.664	103	18.31	81.69
**POPC/Erg 75/25 (μM)**									
100	36.9		2.40			0.695			
200	36.9	32.6	2.40	0.0372	0.65	0.695	44.9	35.00	65.00
400	36.9	32.6	2.57	0.0251	0.67	0.648	66.4	13.34	86.66
600	36.9	32.6	2.63	0.0234	0.70	0.634	71.1	10.00	90.00
800	36.9	32.6	2.63	0.0240	0.73	0.634	69.5	8.00	92.00
1000	36.9	32.6	2.63	0.0155	0.75	0.634	108	7.42	92.58

REFERENCES

(1) Guimarães, T. Z. A.; Mello, É. O.; Lucas, D. R.; Damica, F. Z.; Magalhães, F. S. S.; Basso, L. G. M.; Carvalho, A. O.; Gomes, V. M.; Taveira, G. B. *In Vitro* and *In Vivo* Antifungal Efficacy and Safety of the *Ca* Def2.1 G27-K44 Peptide against the Neglected and Drug-Resistant Pathogen *Candida Krusei*. *ACS Bio & Med Chem Au* 2025. https://doi.org/10.1021/acsbiomedchemau.5c00020.
